# Evaluation of the Dye Extraction Using Designed Hydrogels for Further Applications towards Water Treatment

**DOI:** 10.3390/gels10030159

**Published:** 2024-02-21

**Authors:** Magdalena Blachnio, Malgorzata Zienkiewicz-Strzalka

**Affiliations:** Faculty of Chemistry, Maria Curie-Sklodowska University, M. Curie-Sklodowska Sq. 3, 20-031 Lublin, Poland; malgorzata.zienkiewicz-strzalka@mail.umcs.pl

**Keywords:** adsorption kinetics, adsorption equilibrium, dye adsorption, Acid Red 88, chitosan–silica hydrogel, biopolymer, chitosan

## Abstract

In this work, novel chitosan–silica hydrogels were synthesized and investigated by various complementary techniques. The hydrogels were obtained via the immobilization of chitosan (Ch) on the surface of mesoporous cellular foams (MCFs). The latter silica materials were obtained by a sol–gel process, varying the composition of the reaction mixture (copolymer Pluronic 9400 or Pluronic 10500) and the ageing temperature conditions (80 °C or 100 °C). The role of the silica phase in the hydrogels was the formation of a scaffold for the biopolymeric chitosan component and providing chemical, mechanical, and thermal stability. In turn, the chitosan phase enabled the binding of anionic pollutions from aqueous solutions based on electrostatic interaction mechanisms and hydrogen bonds. To provide information on structural, morphological, and surface properties of the chitosan–silica hydrogels, analyses such as the low-temperature adsorption/desorption of nitrogen, small-angle X-ray scattering (SAXS), scanning electron microscopy (SEM), atomic force microscopy (AFM), and Fourier-transform infrared spectroscopy (FTIR) were performed. Moreover, the verification of the utility of the chitosan–silica hydrogels as adsorbents for water and wastewater treatment was carried out based on kinetic and equilibrium studies of the Acid Red 88 (AR88) adsorption. Adsorption data were analyzed by applying various equations and discussed in terms of the adsorption on heterogeneous solid-surfaces theory. The adsorption mechanism for the AR88 dye–chitosan–silica hydrogel systems was proposed.

## 1. Introduction

Hydrophilic properties combined with the limited solubility of hydrogels in the aqueous environment have led to a growing interest and usability in biomedical, pharmaceutical, and environmental applications [1,2,3,4,5]. Hydrogels are three-dimensional networks of polymer chains that can bind large amounts of water and retain the structure of a flexible solid. Hydrogels based on biopolymers are a class of such materials that have attracted considerable attention in recent years due to their unique properties and potential applications. These hydrogels consist of natural or synthetic polymers that can absorb large amounts of water and swell into a gel-like material. Biopolymer-based hydrogels have many advantages over synthetic hydrogels, including biodegradability, low toxicity, and easier accessibility. The anhydrous hydrogel structure can reabsorb large amounts of water and rebuild itself into a unique three-dimensional structure due to its hydration and the presence of capillary regions. The presence of water in the structure of hydrogels acts as a plasticizer and increases the flexibility of the polymer or biopolymer chains. The high water content in hydrogels allows for the diffusion of different molecules inside and outside their extended porous structure, which is successfully used in the development of systems for controlled adsorption exchange. The internal structure of hydrogel materials depends largely on the chemical structure of the individual monomers and the cross-linking of the polymer chains. The cross-linking ensures the three-dimensional, water-insoluble structure and the physical integrity of the hydrogel. Depending on the cross-linking density, a porous structure with specific sizes and pore densities is obtained.

Due to their good mechanical and thermal stability, their high chemical stability, and their easy recyclability, biopolymer-based hydrogels are effective matrices for environmental protection (e.g., in wastewater treatment). In this context, chitosan functionality is of particular interest due to the properties of the polymer, such as bioactivity, non-toxicity, biocompatibility, biodegradability, and high sorption capacity. An important example of the biopolymer system is chitosan. The presence of hydroxyl and amino groups, the effective ability to adsorb various pollutants (e.g., heavy metals and dyes) through hydrogen bonding and electrostatic interactions, and the possibility to adjust the physicochemical properties are only the most important features of this type of biopolymer. Despite their value, products made only from chitosan, e.g., in the form of membranes, encounter certain limitations, such as relatively low mechanical and chemical strength. This includes increasing mechanical resistance, which must be achieved by chemical cross-linking reactions or in combination with other polymers, usually polysaccharides or inorganic systems. The idea of combining the advantages of hydrogels and chitosan can improve the adsorption capacity and efficiency of chitosan-based hydrogels. The creation of new hydrogel forms and the modification of available solutions are the result of the need for new functional materials. In the world of biopolymer hydrogel systems, active research concerns chitosan-based composite hydrogels for biomedical applications [6,7,8,9,10,11] such as novel heat-sensitive chitosan hydrogels in combination with polyols or polyoses [12]; gold and silver–gold chitosan hydrogels and hydrogel-modified textiles [13,14]; supramolecular chitosan-based hydrogels for 3D bioprinting [15,16]; hydrogels as space-confining media for the synthesis of nanostructural materials [17]; superporous chitosan hydrogels as pH-sensitive materials [18]; and chitosan-based hydrogels for multifunctional, wearable sensory properties [19]. Final chitosan-based hydrogels have gained considerable attention in terms of water purification due to their great adsorption capacity toward pharmaceutics, heavy and rare earth hazardous metals [20,21], and dyes [22,23,24]. Attempts to strengthen the biopolymer network include many ideas and technological solutions. Examples include the addition of nanographene oxide, which significantly reduces the storage modulus of the cross-linked network, which is very detrimental to mechanical performance [25], and the preparation of homogeneous composite films with improved mechanical properties [26,27]. To improve the mechanical properties of chitosan hydrogels and create highly porous membrane systems, the porous phases of silica can be used.

To fully assess the spectrum of usefulness of chitosan–silica materials, certain limitations of both components should be taken into account. These include the following: (i) the adsorption properties, which depend on the sources of the biopolymer and their quality; (ii) various stages of heterogeneity related to the distribution of the acetyl groups along the backbone; (iii) limited surface area and porosity; and (iv) the moderate stability of chitosan materials for long-term applications [28]. The limitation of silica is its surface, which is poor in functional groups and has no significant affinity for molecules such as dyes. The effectiveness of chitosan and chitosan derivative materials for interaction with dyes has been studied by various researchers. Some of them include the application of chitosan composites for the removal of reactive dyes (reactive red 222, reactive blue 222, and reactive yellow 145 [29]; reactive black 5 [30]; and many others).

In the evaluation of adsorbents intended for the removal of anionic dyes, many researchers have used Acid Red 88 (AR88) as a model dye from the group of sulfonated azo compounds [31,32,33,34,35,36,37]. This group of dyes is widely used in various industries (textile, leather, plastics, paper, pharmaceutical, food, and cosmetics industries) [38,39,40], implying a high probability of water pollution with the mentioned substances. Due to the complexity of the structural composition of sulfonated azo compounds (one or more azo groups having aromatic rings substituted by sulphonate groups), their degradation by chemical or biological decomposition is limited [40,41,42], which makes the method based on the adsorption phenomenon superior to others. Moreover, their high solubility in water and unique chemical structure are responsible for the intense color, decreasing light penetration within water bodies. As a result, the photosynthetic activity of plants is inhibited, the amount of dissolved oxygen decreases, and the conditions for fauna deteriorate.

Currently, activated carbon is commonly used as an adsorbent in on-site sewage treatment plants; however, its microporous characteristics make the utility of this material insufficient for high-molecular pollutants due to slow kinetics [43]. Therefore, there is a growing need to find an effective mesoporous alternative for the removal of larger dyes from effluents. The development in the field of new mesoporous materials combining chitosan and silica phases is the response to the challenge. The mesoporous structure of chitosan–silica composites/hydrogels enables fast pollutant diffusion in their internal space, which significantly shortens the time for reaching adsorption equilibrium. It should be emphasized that in the decolorization process conducted in large-scale technological systems, kinetics is as important as the adsorption capacity of the material used.

A number of materials such as surfactant-modified bentonite [31], chitosan–silica gel composite [32], chitosan–nanosilica composite [33], magnetic ZnFe_2_O_4_ spinel ferrite nanoparticles [34], ZnO/ZnMn_2_O_4_ nanocomposite [35], magnetic MWCN-Fe_3_C composite [36], and zeolite–chitosan hydrogel [37] were employed as adsorbents for AR88 dye removal. However, only mesoporous materials enable high efficiency in pollutant adsorption, taking into account both the kinetics and adsorption capacity of the material.

In this article, the preparation, characterization, and adsorptive properties of chitosan–silica hydrogels with an advanced mesoporous structure are investigated to determine if they are an effective material for adsorption applications aimed at removing high-molecular dyes from aqueous solutions.

## 2. Results and Discussion

### 2.1. Materials Characterization

The analysis of the porosity of hydrogels involves determining the number of voids present in the hydrogel network. This can be achieved by gas sorption at a temperature of liquid nitrogen (77 K) and SAXS analysis. The porosity of hydrogels is an important parameter, as it influences the swelling behavior, adsorbate release, and mechanical properties. Therefore, the porosity analysis is a crucial step in the characterization of hydrogels for their intended application.

**Figure 1:**
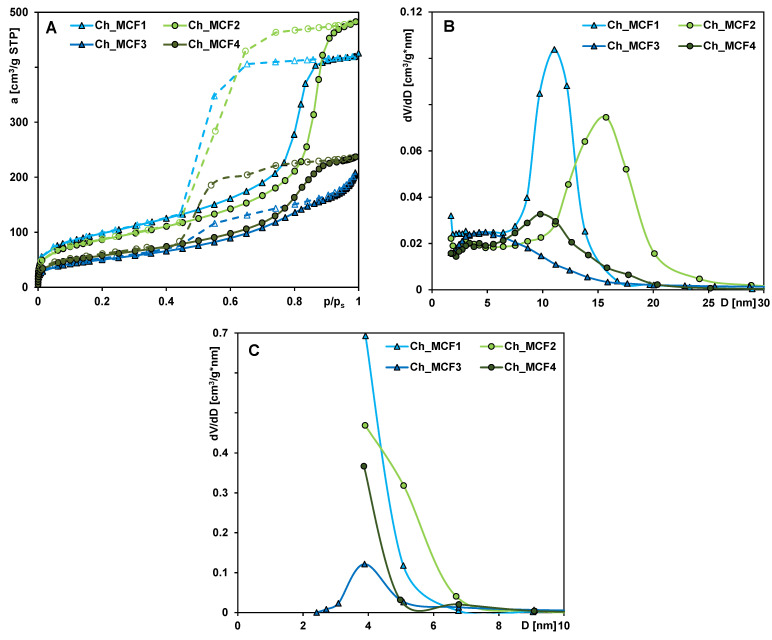
(**A**) Nitrogen adsorption–desorption isotherms at 77 K of the analyzed samples, (**B**) porosity distributions calculated with the BJH theory from the adsorption, and (**C**) desorption branches of the isotherms.

To determine the porosity properties of chitosan–silica hydrogels based on nitrogen adsorption–desorption measurements, the obtained data were used to calculate the isotherms (Figure 1A); the pore size distribution (PSD) functions from the Barrett–Joyner–Halenda (BJH) method (Figure 1B,C); and the porosity parameters, i.e., the specific BET surface area (S_BET_), the external surface area (S_ext_), the total pore volume (V_t_), the micropore volume (V_mic_), the average hydraulic pore diameter (D_h_), the average BJH adsorption pore diameter (D_BJH ads_), and the average BJH desorption pore diameter (D_BJH des_) (Table 1).

The shape of the nitrogen adsorption/desorption isotherms shows that chitosan–silica hydrogels are mesoporous materials with a well-developed hysteresis loop. According to the IUPAC classification, the isotherms are of type IV with an H2 (the Ch_MCF1, Ch_MCF2, and Ch_MCF4 samples) and H4 hysteresis loop (the Ch_MCF3 sample). In the range of low relative pressures, gas adsorption is negligible, which proves the almost complete absence of micropores in the porous structure. When p/p_0_ is close to 0.4, a hysteresis loop is formed, and after exceeding the value of p/p_0_ = 0.7–0.8, a clear increase in gas adsorption can be seen, which corresponds to capillary condensation in pores in the range of 2–50 nm. Wide hysteresis loops indicate the presence of pores with narrow entrances and wide interiors (i.e., bottle-shaped pores). For the Ch_MCF1 and Ch_MCF2 samples, a significant increase in the slope of the adsorption branch of the isotherm in a narrow range of relative pressure indicates that these materials are characterized by the most homogeneous pore structure. This is confirmed by the pore distribution (PSD) functions determined from the adsorption branch of the isotherm, where clearly defined peaks can be seen in the region corresponding to the average size of the mesopores. For the Ch_MCF4 sample, only a slight increase in nitrogen adsorption is observed at a moderate and high relative pressure (with a gentle slope), indicating a much less pronounced mesoporosity with the presence of pores with a wide range of diameters. The PSD spectrum is quite broad, flattened, and bimodal (dominated by small- and medium-sized mesopores). However, the Ch_MCF3 sample is found to have the least amount of gas uptake across the range of relative pressures and different shapes of the hysteresis loop, indicating that this hydrogel is characterized by the least development of the porous structure with the slit-shaped mesopores.

According to the data presented in Table 1, chitosan–silica hydrogels have a specific surface area, ranging from 181 to 351 m^2^/g. The adsorption–desorption data show that a significant part of the specific surface area corresponds to the external surface area (typical for solids whose porosity is limited to mesoporosity). Significant differences in the size of the specific surface area of hydrogels are related to differences in the values of their total pore volume (V_t_ = 0.31–0.75 cm^3^/g) and average pore size (D_h_ = 7–10 nm; D_BJHads_ = 7.2–10.3 nm; D_BJHdes_ = 4.2–5.7 nm). Significant differences between the average BJH adsorption pore diameter (D_BJHads_) and the average BJH desorption pore diameter (D_BJHdes_) can be explained by difficulties in gas desorption from pores with narrow entrances and wide interiors (bottle-shaped pores). This can also be recognized by the incomplete outline of the PSD determined from the desorption branch of the isotherm. Only in the case of the Ch_MCF3 sample, the values of D_BJHads_ and D_BJHdes_ do not differ significantly, and PSD_des_ is completely scratched, which is related to the presence of slit-shaped pores. Analyzing the porosity properties of the final hydrogels and the conditions for the synthesis of the silicate carrier phases (ageing temperature 80 °C or 100 °C), it can be seen that samples obtained from silica aged at a lower temperature (Ch_MCF1 and Ch_MCF2) have a larger specific surface area and total pore volume than samples based on silica processed at higher temperatures (Ch_MCF3 and Ch_MCF4). When varying the composition of the reaction mixture used to synthesize the mesoporous cellular foams (copolymer Pluronic 9400 or Pluronic 10500), no regularity was observed in the changes in the structural parameters of the final materials.

**Figure 2:**
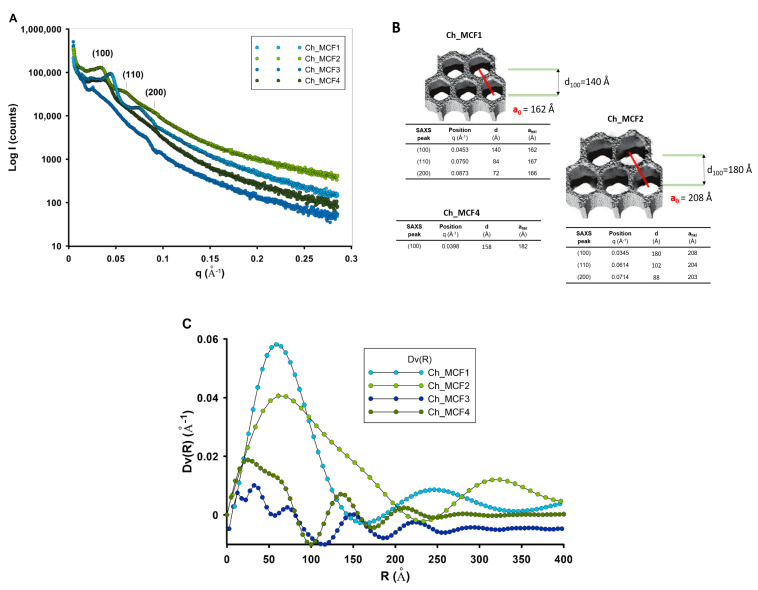
(**A**) SAXS patterns of the investigated hydrogels, (**B**) models of the pore structure of the hexagonal network and structural parameters calculated from SAXS curves, and (**C**) volume-weighted scattering object (pore) size distribution Dv(R) of all investigated hydrogels.

The scattering pattern (by SAXS) provides information about the structure of the material, including the size and shape of the pores, as well as the unit of periodicity of the atomic arrangement (Figure 2A). The SAXS pattern of the Ch_MCF1, Ch_MCF2, and Ch_MCF4 materials shows a series of reflections corresponding to the hexagonal pore structure. One intense peak for the (100) lattice plane was observed for three samples, i.e., Ch_MCF1, Ch_MCF2, and Ch_MCF4, and two additional low-intensity peaks representing the (110) and (200) planes, characteristic of the long-range ordered 2D hexagonal (p6mm) mesostructure, for only two samples of the whole group (Ch_MCF1 and Ch_MCF2). No reflexes are observed for the Ch_MCF3 sample, indicating a lack of structural order.

These data clearly show that a higher ageing temperature of the support disrupts the periodic organization of the pore spaces, and this is directly reflected in a lack of structural order in the hydrogel material. The analysis of the dimensions of the unit cell of the porous structure for which its value could be determined (three samples for which SAXS reflections were observed) (Figure 2B) also suggests that the distance between two neighboring pore centers was larger (~21 nm) when using the pore-forming matrix PE9400 than with PE10500. However, it cannot be excluded that the periodicity perturbations in the Ch_MCF4 sample or their absence (as in the Ch_MCF3 sample) could be related to the local agglomeration of the chitosan phase.

The values describing the structural parameters, especially the lattice parameter a_0_ given in Figure 2B, make it possible to compare these values with the pore size (based on the BJH adsorption data or the size distribution of the scattering particles determined in Figure 2C) and, on this basis, to assess the thickness of the walls between the pores and thus compare their value to the thickness of the biopolymer layer. It is worth noting that the pore sizes of the tested hydrogels, which were determined using two different techniques, correlate quite well with each other. In the case of the Ch_MCF1, Ch_MCF2, Ch_MCF3, and Ch_MCF4 samples, the pore size determined from the adsorption data of the BJH model indicates pore sizes of 7.4, 10.0, 7.0, and 7.1 nm, respectively. The volume-weighted pore size distributions determined from the maximum function of the SAXS data show sizes of ~7 nm, ~10 nm, and ~6 nm for the Ch_MCF1, Ch_MCF2, and Ch_MCF4 samples, respectively. Thus, the difference in unit cell dimensions and pore sizes is ~9.2 nm, 11 nm, and 12 nm for these samples, respectively. The values obtained are consistent with the nitrogen content data and may reflect the size of the biopolymer layer in the final hydrogel materials.

To determine the quantitative content of the organic component in chitosan–silica hydrogels, the Ch_MCF1, Ch_MCF2, Ch_MCF3, and Ch_MCF4 samples were analyzed for the elemental composition of the basic elements (nitrogen, hydrogen, and carbon). The results of these measurements are summarized in Table 6 (Section 4.2. Synthesis of Materials). Based on the analysis of the samples, it was found that the chitosan content in three samples (Ch_MCF2, Ch_MCF3, and Ch_MCF4) is comparable, while it is significantly lower in one sample (Ch_MCF1). It is assumed that this deviation is due to incomplete dissolution of the chitosan during the process of silica impregnation or to the deposition of a layer of the biopolymer film on the walls of the reaction vessel. The total amount of nitrogen in hydrogels is related to the amount of amino and amide groups from the organic phase, which determines the adsorption capacity of these materials towards anionic pollutants. As a rule, a larger amount of nitrogen groups should have a better effect on the adsorption capacity of materials. On the other hand, it must be noted that the degree of utilization of the organic phase as the component responsible for adsorption depends on its availability for the adsorbate. Therefore, when analyzing the adsorption capacity of chitosan–silica hydrogels, both the amount of chitosan and its distribution in the structure of the silicate carrier are taken into account.

**Figure 3:**
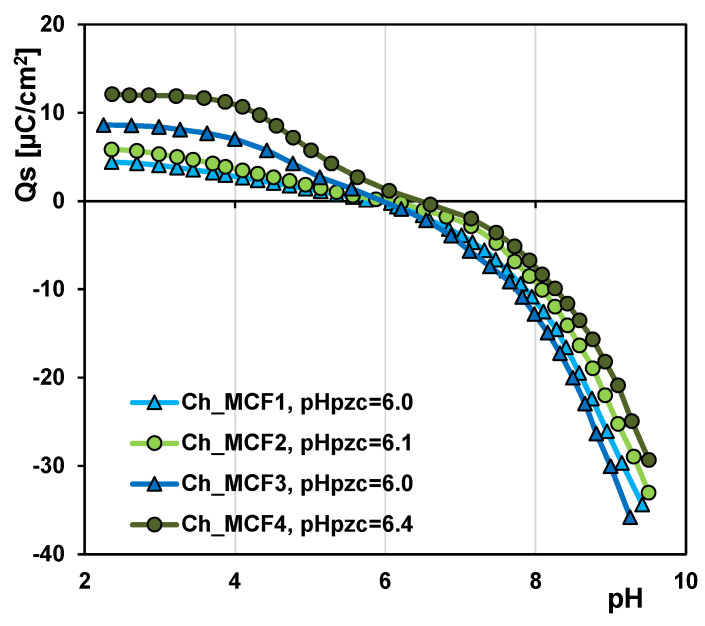
Dependences of surface charge densities of chitosan–silica hydrogels on solution pH.

Based on the potentiometric titration data of chitosan–silica hydrogels, the dependences of surface charge densities on a solution pH were graphically plotted (Figure 3). The pH values of zero charge point (PZC) were defined as 6.0, 6.1, 6.0, and 6.4 for Ch_MCF1, Ch_MCF2, Ch_MCF3, and Ch_MCF4, respectively. One can say that the PZC values are similar; only for Ch_MCF4, it is slightly higher, which can be related to the highest content of the chitosan component in this sample. Due to the presence of silanol groups, pure silicate molecular cell foams (MCFs) are characterized by an acidic nature, and the PZC value is in the range of 4–5.6 [44]. Generally, the combination of a silica carrier with a chitosan phase of a polycationic nature leads to a change in the acid–base properties of the final materials compared to pure MCFs.

**Figure 4:**
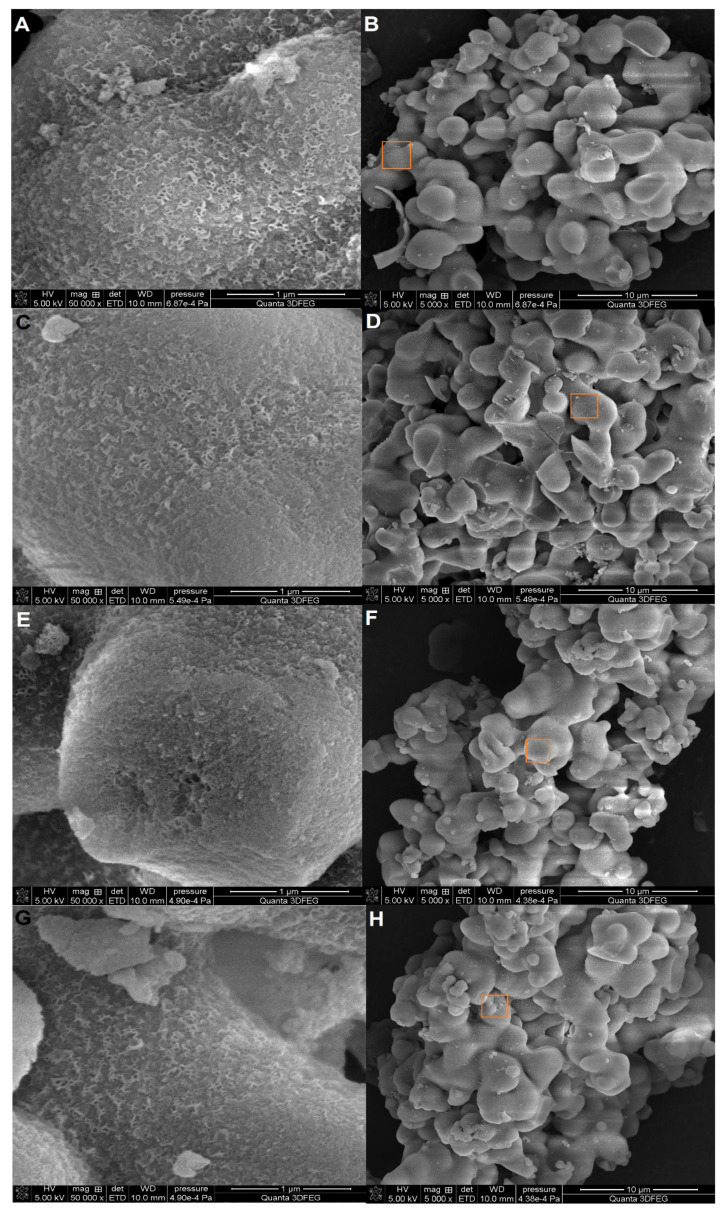
SEM images of the analyzed materials at two selected magnifications: Ch_MCF1 (**A**,**B**), Ch_MCF2 (**C**,**D**), Ch_MCF3 (**E**,**F**), and Ch_MCF4 (**G**,**H**). The marked fragments are enlarged in the left images.

The morphological analysis using scanning electron microscopy (Figure 4A–H) revealed that the surface of the samples had a highly porous structure with interconnected pores of different sizes and shapes. The surface of the silica foams appeared to be rough and irregular, with some areas having a smooth texture. The images also showed that the silica foams had a large surface area, which is desirable for adsorption applications. Overall, the SEM images of the silica foams showed their unique and desirable structural properties for various applications, which is consistent with the structure of the chitosan–silica hydrogels. These results indicate that the chitosan–silica hydrogels have high surface area and porosity, which can improve their adsorption capacity.

The SEM images make it possible to observe surface changes due to the modification of the chitosan phase and to correlate them with the content of this phase by the percentage of nitrogen following the elemental analysis carried out (Table 6). In the case of the Ch_MCF1 sample, where the nitrogen content is the lowest (0.8%), the morphology of the hydrogel is the most homogeneous. The SEM images taken for this sample (even at lower magnifications, showing a larger area, which are not included here) show no visible agglomerates of the chitosan phase. This bottom structure, where the chitosan layer most likely forms a thin surface layer supported by the extended structure of the porous silica, is directly reflected in the uniformity of the distribution of this porous structure already mentioned when analyzing the nitrogen adsorption/desorption data. For comparison, in the Ch_MCF4 sample, where the highest chitosan content is expected (corresponding to the highest percentage of nitrogen (1.2%)), the SEM images show localized areas of aggregation of the biopolymer phase visible as white fragments differentiating the hydrogel surface. This is reflected in the disturbance of the uniform pore distribution, as it may block access to the porous structure of the silicate support. Therefore, such a localized increased presence of chitosan may lead to some increase in dye adsorption capacity. The SEM images of the Ch_MCF2 and Ch_MCF3 samples do not differ significantly morphologically, although some signs of agglomeration of the chitosan phase can be seen.

**Figure 5:**
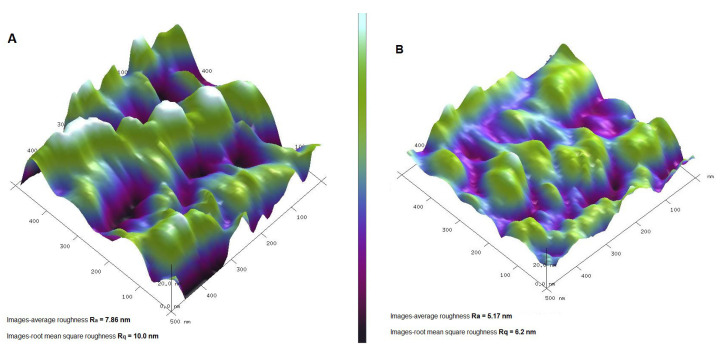
Atomic force microscopy (AFM) topography images of Ch_MCF1 (**A**) and Ch_MCF4 (**B**).

The imaging of the analyzed samples was complemented by an AFM analysis performed on powder samples that made it possible to assess the topography of the material considering three dimensions (including the z-dimension). The surface texture of two selected samples (Ch_MCF1 and Ch_MCF4) was chosen for AFM analysis, as the largest differences in nitrogen content (0.8% for Ch_MCF1 and 1.2% for Ch_MCF4) indicate differences in the amount of the chitosan component (Figure 5). Analyzing the surface roughness of selected systems therefore made it possible to confirm the differentiation of the surface depending on the amount of biopolymer phase. The surface of the Ch_MCF1 sample was rougher (R_a_ = 7.86 nm and R_q_ = 10 nm) than that of Ch_MCF4 (R_a_ = 5.17 nm and R_q_ = 6.2 nm). The results obtained are consistent with other methods indicating that the chitosan biopolymer can form a thin layer on the surface covering the substrate. It should be noted that this conclusion only applies to areas where the biopolymer is homogeneously distributed. Areas in which agglomeration of the biopolymer phase can occur cannot be ruled out. This is a problem that is difficult to eliminate, and such a phenomenon should be taken into account, especially in systems containing even larger amounts of biopolymer.

**Figure 6:**
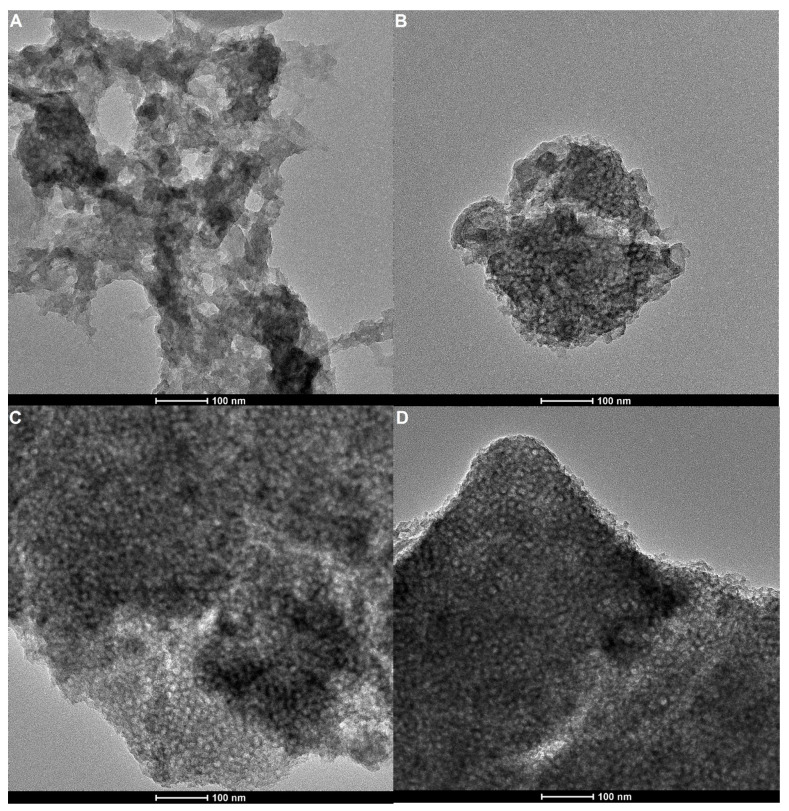
TEM images of the analyzed materials: Ch_MCF1 (**A**), Ch_MCF2 (**B**), Ch_MCF3 (**C**), and Ch_MCF4 (**D**).

The tested materials were imaged using TEM microscopy (Figure 6). The recorded TEM images made it possible to visualize the mesostructural nature of the silica carrier, whose characteristic feature is a wide-porous silica network. The biopolymer (chitosan) phase forms a thin layer on the surface of the silica carrier. No significant differences were identified between individual materials.

### 2.2. Adsorption at Equilibrium State

To evaluate the practical utility of the obtained chitosan–silica hydrogels for environmental protection, these materials were tested as adsorbents in the process of adsorption purification of aqueous solutions from potential pollutants. The chosen water purification method, which utilizes the physical or chemical adsorption of polluting substances on a solid, is considered to be highly effective, selective, cost-effective, and energy-saving. In addition, no chemicals are used in this process and no by-products are produced that could become secondary pollutants. The proposed adsorbents (chitosan–silica hydrogels) are based on a biodegradable polymer—chitosan and environmentally neutral silica—which means that the purification process using them meets the requirements of sustainable technology. The physicochemical and adsorption properties of the hydrogels’ components, i.e., chitosan and silica, were decisive for the selection of an anionic adsorbate, i.e., Acid Red 88 (AR88). It was assumed that silica in the form of a mesoporous cellular foam would provide a scaffold for the biopolymer and improve the thermal and mechanical resistance of the final product but would not itself be involved in binding organic pollutants. Chitosan is characterized by a polycationic nature and has a high capability towards anionic compounds, so the adsorption capacity of hydrogels for AR88 is solely due to the presence of the organic phase.

**Figure 7:**
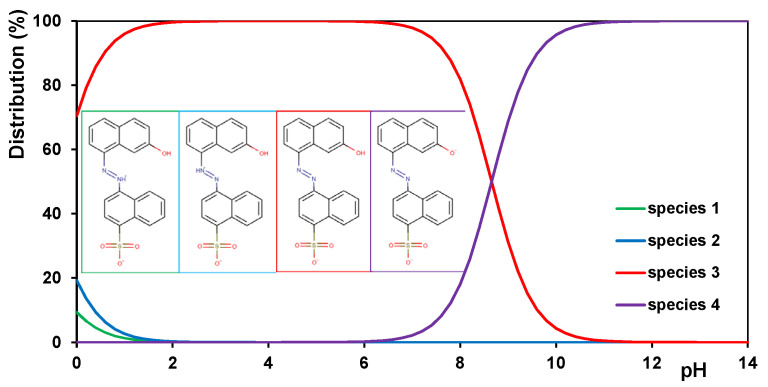
Distribution of the possible molecular forms of the dye AR88 depending on the solution’s pH.

In Table 2 and Figure 7, the physicochemical parameters of AR88 and the distribution of its possible molecular forms depending on the solution pH are presented, respectively.

**Figure 8:**
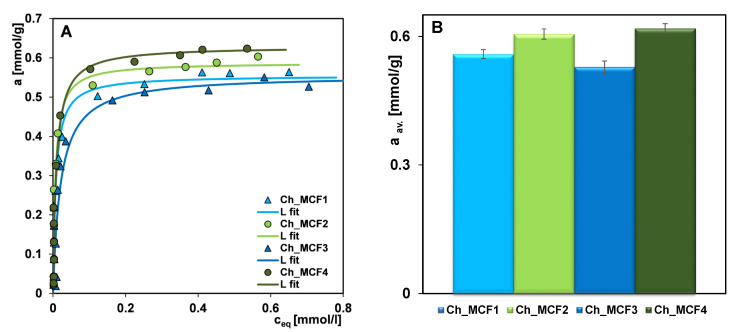
(**A**) Adsorption isotherms of Acid Red 88 on chitosan–silica hydrogels. (**B**) Bar graph of the average amounts of dye adsorbed onto chitosan–silica hydrogels calculated from four adsorption trials, along with error statistics (m = 0.05 g; c_0_ = 1.96 mmol/L; V = 0.025 dm^3^).

Figure 8A shows the results of the adsorption of Acid Red 88 on chitosan–silica hydrogels, with fitting curves using the Generalized Langmuir equation (more precisely, its simpler form—the Langmuir isotherm) [48]. To evaluate the consistency of the amounts of dye adsorbed on the tested chitosan–silica hydrogels, the results of four adsorption trials, along with the error statistics, are graphically depicted in Figure 8B. The highest standard deviation was obtained for the Ch_MCF3 hydrogel (±2.6%), while the values for the other samples were less than ± 2%. The relatively small dispersion of the adsorption measurement results in their average value results from the high quality of all adsorbents and the maintenance of the constant conditions of the experiment. This, in turn, confirms the reliability of the presented results and the possibility of the effective use of hydrogel materials for industrial or real-life applications.

The maximum adsorption capacity (a_m_) and the adsorption constant (log K) for the Ch_MCF1, Ch_MCF2, Ch_MCF3, and Ch_MCF4 hydrogels are as follows: 0.56, 0.59, 0.55, and 0.63 mmol/g; and 2.12, 2.18, 1.68, and 2.04, respectively (Table 3). The analysis of the a_m_ values and elemental composition of each adsorbent suggests that the adsorption efficiency is largely dependent on the nitrogen content in the hydrogels as an elemental component of the amino and amide groups (adsorption active centers of the organic component), but there are undoubtedly other factors that should be considered. The close correlation between a_m_ and solid composition is visible for the Ch_MCF4 hydrogel. Meanwhile, Ch_MCF3 with a nitrogen content comparable to that of Ch_MCF2 and Ch_MCF4 has the weakest adsorption capacity, while Ch_MCF1 with the lowest nitrogen content works as an adsorbent at the level of the Ch_MCF3 hydrogel. High log K values (2.04–2.18) indicate a strong affinity of the dye to the hydrogels and a high strength of their mutual interactions. This can be explained by the bottle-like shape of the pores of the adsorbents, as it hinders the possible desorption of the adsorbate. The exception is the AR88/Ch_MCF3 system, for which log K = 1.68. Here too, the weaker affinity and the lower strength of the interactions between adsorbate and adsorbent can be linked to the type of adsorbent pores (slit-shaped pores).

**Figure 9:**
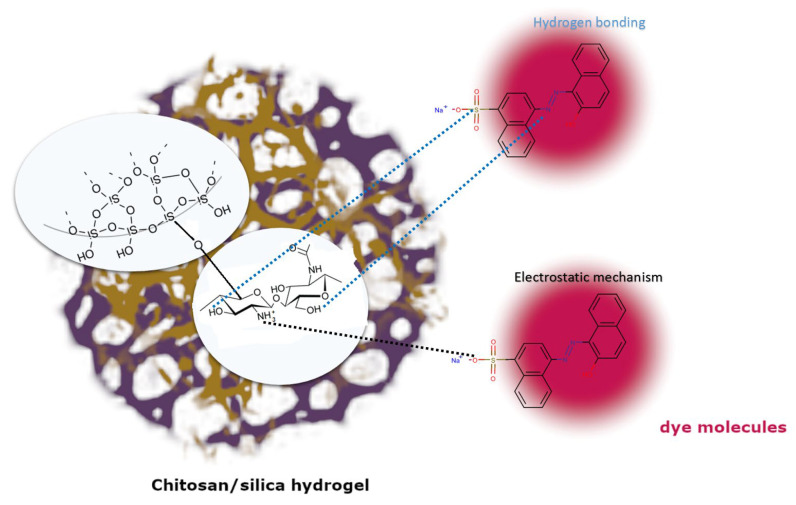
Schematic diagram of adsorption mechanism at the molecular level.

Due to the polycationic nature of chitosan, the main mechanism of dye adsorption on chitosan–silica hydrogels is based on electrostatic interactions between protonated amino groups and dye anions. In addition, the presence of hydrogen bonds between hydroxyl, azo, sulfone, and hydroxyl groups originating from the adsorbate or adsorbent is very likely. The silica phase of the hydrogels did not participate in the adsorption of the anionic dye due to the lack of mutual affinity, which was experimentally confirmed in the paper [33]. A schematic diagram of AR88 adsorption mechanism at the molecular level is presented in Figure 9.

The adsorption mechanism of the dye can change depending on the chemistry of the solution (e.g., pH and ionic strength) and the adsorption temperature, which consequently affects the adsorption efficiency. When the pH of the solution changes, both the degree of ionization of the dye molecules (Figure 7) and the surface charge of the hydrogels (Figure 3) are affected. At an acidic pH, the degree of protonation of the amino groups of chitosan is highest, so the strongest attractive interactions between the adsorbent and adsorbate are to be expected. Under neutral conditions, only some of the amino groups remain in the protonated form, weakening the electrostatic interactions in the adsorption system. In the proposed method for synthesizing hydrogels, the phase of cross-linking the chitosan phase, which is usually carried out with environmentally harmful substances such as formaldehyde or glutaraldehyde, is omitted. Therefore, the adsorbents obtained are not suitable for use under acidic conditions in which the biopolymer phase can dissolve without additional modification. It should be emphasized that the synthesis approach without cross-linking of the biopolymer phase decreases the stability of materials under acidic conditions but simultaneously means their cheaper production, environment sustainability, and easier disposal. Both hydrogel phases are environmentally friendly: chitosan is because of its biodegradability; and silica is because it is neutral to the environment, a natural component of the earth’s crust, sand, and rocks.

In describing the factors influencing the performance of the adsorbents, their structural properties were analyzed. All hydrogels are typical mesoporous materials with a pore size larger than the size of the adsorbate molecules. Therefore, the effect of dye exclusion in the adsorption process can be neglected. Regarding the degree of development of the specific surface area and total pore volume of the hydrogels, no clear correlation between porosity and dye removal efficiency was observed. The Ch_MCF1 and Ch_MCF3 hydrogels with different structural parameters (S_BET_ = 351 m^2^/g and V_t_ = 0.65 cm^3^/g; and S_BET_ = 181 m^2^/g and V_t_ = 0.31 cm^3^/g, respectively) work as adsorbents at a similar level. However, the Ch_MCF4 hydrogel with relatively low porosity (S_BET_ = 206 m^2^/g and V_t_ = 0.36 cm^3^/g) shows the highest adsorption of the pollutant.

**Figure 10:**
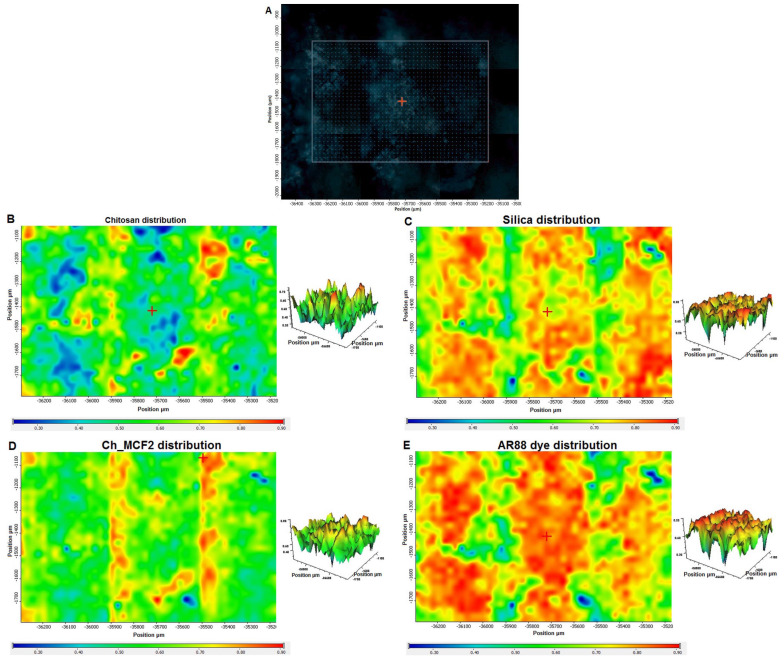
Correlation map of the components on AR88/Ch_MCF2: (**A**) microscopic image of the mapped area 1150 µm × 750 µm (the dots represent the measured FTIR spectra); distribution correlation map of (**B**) chitosan, (**C**) silica, (**D**) Ch_MCF2 hydrogel support, and (**E**) AR88 dye.

The degree of coverage of the silica with a chitosan layer and its probable forms were also investigated. The optimal adsorption capacity of chitosan–silica hydrogels seems to be reached when the organic phase (chitosan) completely covers the outer and inner surfaces of the silica and has the form of a homogeneous and thin film. In such a case, the porosity of the silica is of crucial importance, as it ensures the maximum utilization of the active adsorption sites of the organic phase. The most unfavorable hydrogel structure for the adsorption of pollutants, on the other hand, is one that has only partial coverage of the silica with a biopolymer in the form of thick, compact fibers formed by the agglomeration of its macromolecules. Such an adsorbent provides only limited access to the active sites for the adsorbate and does not support effective adsorption. There are also possible structures that are a combination of the above variants, and this type of structure is represented by the chitosan–silica hydrogels, which are the subject of our research. To explain this phenomenon, we used FTIR microscopy analysis and surface mapping of the exemplary material. FTIR mapping is a powerful analytical technique used for chemical imaging that enables the identification and localization of chemical compounds within a sample area. By measuring the absorption and transmission of infrared light, FTIR mapping creates detailed images that can be used to determine the chemical composition of a sample. For example, the quality of coverage and the composition of the dye/hydrogel material (AR88/Ch_MCF2) was analyzed using FTIR microscope mapping. Figure 10B–E show the distribution of the chitosan, silica, chitosan–silica hydrogel, and dye components, respectively, on the sample area of 1150 µm × 750 µm. The dye distribution map (Figure 10E) shows that practically the entire area falls within the range of medium and high intensities of the measured signal (colors from green to red), proportional to the amount of adsorbed dye.

Areas with low signal intensity (dark blue and blue) are residual. Silica has no adsorption capability for the dye, only chitosan, which suggests that practically the entire surface of the silicate carrier is covered with the biopolymer. In turn, analyzing the maps of chitosan (Figure 10B) and dye (Figure 10E), it can be seen that the areas of medium and high signal intensity (yellow and red, respectively) coming from the biopolymer do not coincide with the areas with a relatively large amount of adsorbed dye. There is an opposite tendency, indicating that a thin layer of the biopolymer (navy blue and blue areas) favors the binding of the pollutant compared to fibrous forms that hinder access to the adsorption-active centers (yellow and red).

**Figure 11:**
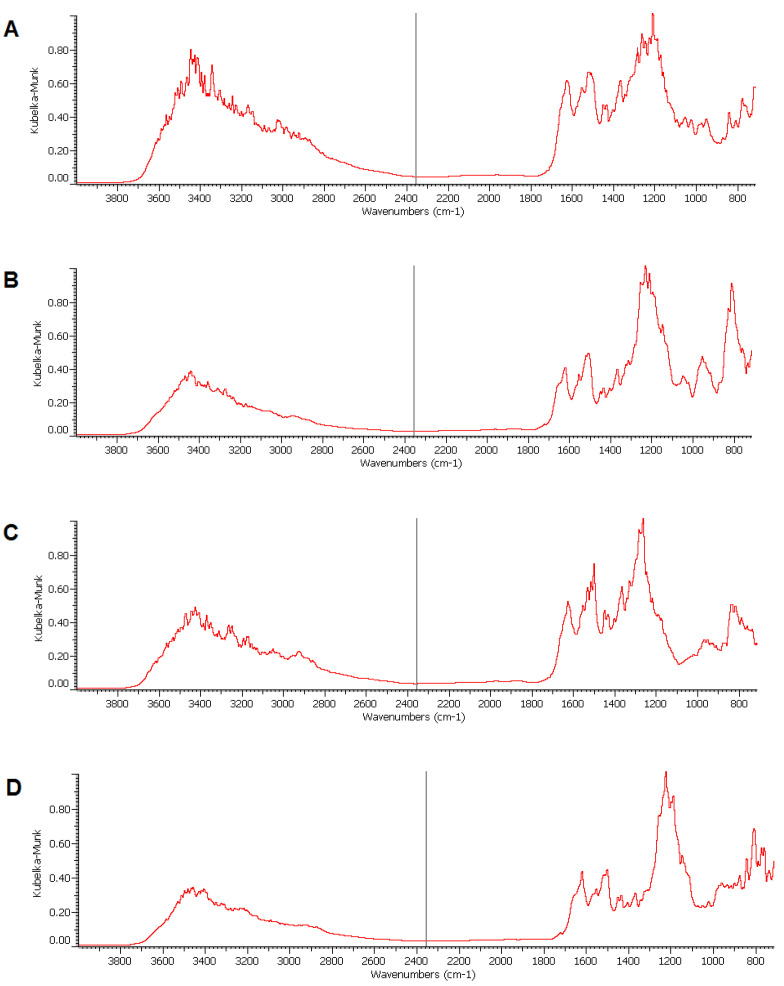
The FTIR spectra of the corresponding maps represent the point marked with a red cross for distributions of (**A**) chitosan, (**B**) silica, (**C**) Ch_MCF2 hydrogel support, and (**D**) AR88 dye.

To control the analyzed areas of the FTIR maps, the FTIR spectrum was determined from a characteristic point (red cross on the correlation maps for the tested systems). In the case of the analysis of the chitosan decomposition (Figure 11B), the control spectrum shows, among other things, the bands of OH and NH stretching at 3440 cm^−1^, and the absorption band at 2880 cm^−1^, which originates from the stretching vibrations of the C–H bond in this polysaccharide structure. In addition, the stretching vibrations of the C=O group at 1640 cm^−1^, the N-H group at 1570 cm^−1^, the C-OH group at 1380 cm^−1^, and the C-O-C group at 1200 cm^−1^ can be recognized. All of these group vibrations confirm the structure of chitosan in the tested system. The FTIR spectrum of the silica component covered by the map in Figure 11C shows the presence of signals typical of silica material, namely stretching vibrations of -OH groups at 3400 cm^−1^, Si-O H vibrations at 1540 cm^−1^ and 950 cm^−1^, and Si-O-Si at 1000–1200 cm^−1^ and 800 cm^−1^. In the hydrogel/dye system, the FTIR spectrum confirms the presence of groups characteristic of the dye molecule. AR88 has the two most characteristic bands at 3415 cm^−1^ and 1618 cm^−1^ for the OH– and N=N groups, respectively (Figure 11D). The aromatic C=C ring vibrations appear at 1500 and 1550 cm^−1^, while the peaks at 1200 and 1086 cm^−1^ originate from the vibrations of the SO_3_ groups. Finally, the C–H (in-plane) and C=C (out-of-plane) bending vibrations of the aromatic form were identified at 700–850 cm^−1^.

It can be concluded that the adsorption performance of chitosan–silica hydrogels towards Acid Red 88 depends on several factors, namely (i) the characteristics of the porous structure, (ii) the contribution of chitosan and silica, (iii) the degree of coverage of the silica matrix with the chitosan layer, and (iv) the structural form of the organic phase. The adsorption performance of the proposed hydrogels towards AR88 is satisfactory compared to that of other materials presented in the literature (Table 4). The maximum adsorption capacity for Ch_MCF4 is even better than many composites or modified clay.

Additionally, the cost–benefit of the selected adsorbents (presented in Table 4) was evaluated based on the comparison of their adsorption capacity towards dye adsorbates in regard to the synthesis cost, the impact on the natural environment (energy consumption, harmfulness of reagents used, and secondary waste), and their recyclability. The presented evaluation is subjective; however, it is accepted and practiced by many scientists [49,50].

From a practical point of view, it is possible to use industrial chitosan as a cheaper substitute for pure material. This is a way to reduce costs and move adsorbent production to a larger scale. Our research in this area confirmed that the efficiency of dye adsorption by the adsorbent prepared in this way differs only slightly compared to the use of purified chitosan. However, it should be noted that the industrial chitosan material used (e.g., water engineering, a textile finishing agent (to remove dyes from effluent and produce fibers), paper additives (to strengthen the recycled paper and increase moisture resistance), soil improvement, seed treatment (within coatings of slow-release fertilizer pellets and plant diseases control agent), nematode controls, etc.) [51] may contain some impurities that should be considered in the specific application.

### 2.3. Adsorption Kinetics

**Figure 12:**
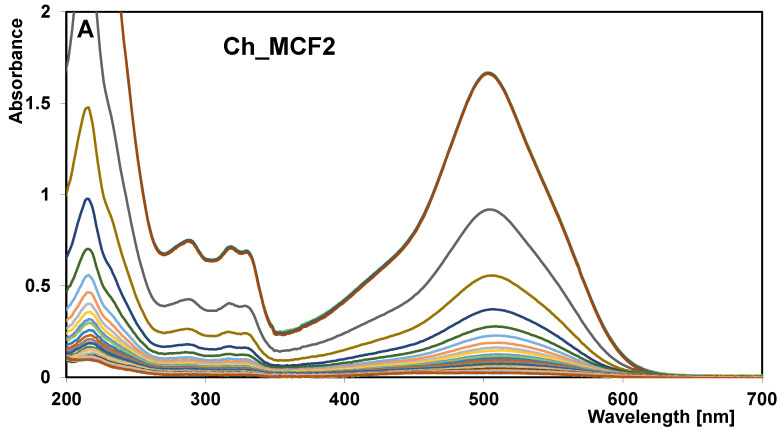

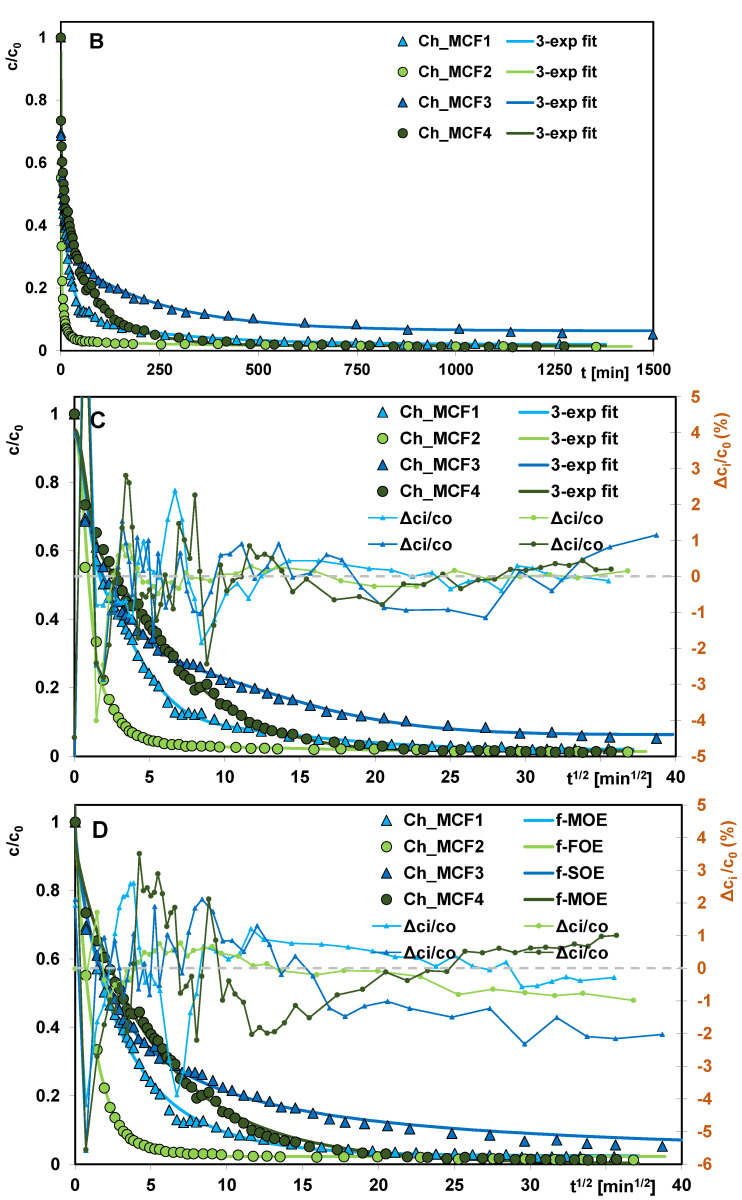
(**A**) A series of UV-Vis absorption spectra obtained for the AR88/Ch_MCF2 system. (**B**) Comparison of the adsorption kinetics of AR88 on chitosan–silica hydrogels at the coordinates: relative concentration~time and (**C**,**D**) concentration~square root of time. The lines correspond to the fitted m-exponential equation (**C**) and fractal-like MOE equation (**D**).

To investigate the adsorption kinetics of AR88 on chitosan–silica hydrogels, measurements of the changes in adsorbate concentration in solution as a function of experiment time were carried out using the technique of continuous absorption spectra recording. A suitably refined method of taking samples from the solution makes it possible to record a series of many spectra (examples in Figure 12A) and to obtain concentration profiles characterized by a large number of points systematically distributed on the time axis (Figure 12B). The course of these curves indicates that the process of dye adsorption on chitosan– hydrogels is very dynamic, especially in the initial phase. This can be seen in the plots of the relative adsorbate concentration (c/c_0_) as a function of the square root of time (t^1/2^) (Figure 12C). The results indicate that the adsorbents took about 1–10 min to bind half of the initial amount of pollutant. The above estimates are based on the half-time values (t_0.5_) obtained by optimizing the experimental data using the multi-exponential equation (m-exp) (Table 5). For the Ch_MCF1, Ch_MCF2, Ch_MCF3, and Ch_MCF4 hydrogels, the parameter t_0.5_ is 4.14, 1.03, 4.85, and 11.23 min, respectively. As the experiment progresses, the process slows down, and significant differences are observed in the values of time corresponding to a certain decolorization efficiency of the solution. For example, the parameters t_75%_ and t_90%_, i.e., the adsorption time to reach 75% and 90% of the process efficiency, range from 2.6 to 70 min and from 10.5 to 440 min, respectively. For three adsorption systems, the equilibrium state is reached after about 200–800 min with a dye uptake (u_eq_) of 98–99%, while for one system (AR88/Ch_MCF3), the time of the experiment (1500 min) is not sufficient to reach the equilibrium state (6% of the dye remains unbound in the solution). The adsorption of Acid Red 88 is fastest on the Ch_MCF2 hydrogel, slightly slower on Ch_MCF1, and slowest on Ch_MCF3 and Ch_MCF4. For the first two adsorbents, the values of the logarithm of the rate constant (log k), which were determined using the m-exp equation, are −0.17 and −0.78, respectively. Such an efficient process on Ch_MCF2 and Ch_MCF1 is undoubtedly related to their large external surface area (S_ext_ = 279 and 318 m^2^/g, respectively), their large total pore volume (V_t_ = 0.75 and 0.65 cm^3^/g, respectively), the low contribution of micropores in the total porosity (1.3–1.5%), and a significant content of mesopores (in the range of D = 10–20 and 8–14 nm, respectively). The latter observation is well illustrated by the pore size distribution (PSD) curves (Figure 1B). For other adsorbents, the adsorption process is more complex as the kinetic curves intersect. Initially, the loss of solute is faster for Ch_MCF3 than for Ch_MCF4, but after reaching the relative concentration = 0.3, the opposite tendency is observed. The values of log k for these adsorbents are −0.84 and −1.21, respectively. The observed trend changes are a consequence of the shape of their pores and the degree of porosity development. The presence of slit-shaped pores in the Ch_MCF3 hydrogel facilitates the free access of the adsorbate to the active centers compared to bottle pores (chambers with narrow openings leading inside) and explains the better efficiency of the adsorption process with this material in the prevailing range of relative concentration. However, the poorer porosity parameters of Ch_MCF3 (S_BET_, S_ext_, and V_t_) compared to those of Ch_MCF4 limit the number of active sites available for the adsorbate. As a result, the process slows down considerably despite the presence of a solute in the solution, which is the driving force of the adsorption process.

Due to the complexity of chitosan–silica hydrogels (porous structure of silica and macromolecular structure of chitosan), an analysis of the kinetic data was performed based on the m-exponential (m-exp) equation and the fractal-like MOE (f-MOE) equation. The m-exponential equation is a fitting function that can be identified with the kinetics of n parallel (and independent) first-order processes or as an approximation to subsequent first-order processes [43]. The fractal-like MOE equation reflects a conception of fractality [52,53]. Both equations assume a certain distribution of adsorption rates and are generally suitable for describing the kinetics of adsorption on heterogeneous solids. Comparing the values of the parameters of the considered kinetic equations, i.e., the logarithm of the rate constant (log k), the adsorption half-time (t_0.5_), the time corresponding to reaching a process efficiency of 75%/90% (t_75%_/t_90%_), and the adsorbate uptake in the equilibrium state (u_eq_), one can observe their high agreement. Similarly, the values representing the quality of the fit of the theoretical models to the experimental data, i.e., the relative deviations (SD (c/c_0_)) and the convergence coefficient (1-R^2^), show compliance (Table 5). It is worth noting that the optimization for the AR88/Ch_MCF2 system results in a value for f_2_ close to zero, so that the f-MOE equation is reduced to its simpler form, i.e., the f-FOE equation.

## 3. Conclusions

Hydrogels are increasingly being researched for their potential applications in water treatment. These three-dimensional networks of hydrophilic polymers can absorb large amounts of water, while retaining their structure. Although the arrangement of the porous structure of hydrogels is important in adsorption studies, it is not the only factor that determines their usefulness. In our work, we analyzed several factors that determine the adsorption process of dye molecules and are part of the complex nature of the silica biopolymer material. Among other things, it was found that the structure of the silica carrier is reflected in the structure of the silica biopolymer hydrogel material. The primary structure of the carrier is determined by the changes in temperature processing during synthesis rather than the nature of the polymer matrix. The proposed synthesis route made it possible to obtain materials with an organized pore structure with a significant size (7–12 nm) that can facilitate the transport of the potential adsorbate into the adsorption network. This is one of the factors that determine the success of the adsorption process. The second is the content and quality of the biopolymer layer, which is also involved in this process. For the materials tested, it was confirmed that the ability to create a uniform thin layer of chitosan on a support with the described structure (extended porous network) determines the adsorption capacity of the material towards AR88 or similar dye molecules. Both the results of the kinetics and adsorption equilibrium showed that chitosan–silica hydrogels could be useful in areas requiring the adsorption of dyes. These include, for example, textile processing and dyeing plants. The adsorbents we propose have a mesoporous structure, which enables faster adsorption of large particles (dyes are quite large particles) compared to standard carbon materials, which are rather microporous.

## 4. Materials and Methods

### 4.1. Chemicals

The non-ionic triblock copolymers Pluronic PE9400 and Pluronic PE10500 as sizing agent and structuring agent were purchased from BAFS (West Port Arthur Road, Beaumont, TX, USA). Pluronic PE9400 is a type of triblock copolymer consisting of a hydrophilic polyethylene oxide (EO) block flanked by two hydrophobic polypropylene oxide (PO) blocks (EO_21_PO_50_EO_21_) with a molecular weight of about 4600 g/mol. The molecular weight of the polypropylene glycol block is 2750 g/mol, and the percentage of polypropylene glycol in the molecule is ~40%. Pluronic PE10500 is a triblock copolymer (composed as EO_37_PO_56_EO_37_) with a molecular weight of approximately 6500 g/mol. The molecular weight of the polypropylene glycol block is 3250 g/mol, and the percentage of polypropylene glycol in the molecule is ~50%. Hydrochloric acid (35–38% pure) and acetic acid (99% pure) were purchased from Polish Chemical Reagents (POCh, Poznan, Poland). The aqueous solutions were prepared in water purified with the Millipore system. The 1,2,4-trimethylbenzene (TMB) isomer (98%) and tetraethyl orthosilicate (TEOS ≥ 99.0%) were purchased from Sigma-Aldrich (Poznan, Poland). Chitosan from shrimp shells with a quality of level QL = 200, with a molecular weight of 190,000 to 370,000 Da, and with a degree of deacetylation of ~75% was purchased from Sigma–Aldrich (Poznan, Poland). Acid Red 88 as a dye adsorbate with a purity of 75% was purchased from Sigma–Aldrich (Poznan, Poland).

### 4.2. Synthesis of Materials

**Figure 13:**
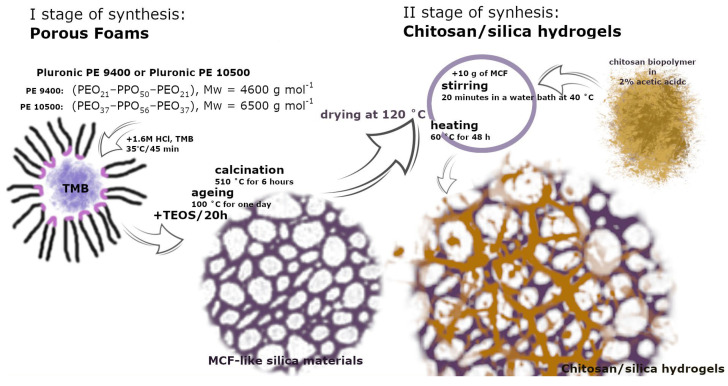
Schematic diagram of the synthesis procedure.

The synthesis of chitosan–silica hydrogels (Ch_MCF1, Ch_MCF2, Ch_MCF3, and Ch_MCF4) is based on the synthesis of porous silica structures in the form of highly porous foams (MCF1, MCF2, MCF3, and MCF4) and presented in Figure 13. These materials formed a scaffold for the biopolymeric chitosan component and offered the possibility of mechanical stabilization and enlargement of the porous surface. The exact amounts of the ingredients can be found in Table 6.

The silica foams (MCF1, MCF2, MCF3, and MCF4) were synthesized by a sol–gel process in which a colloidal suspension of silica nanoparticles in a liquid solvent is converted into a solid foam structure. For each sample, 10 g of polymer (Pluronic PE9400 or Pluronic PE10500) was dissolved in 360 mL of a 1.6 M hydrochloric acid solution. To dissolve the surfactant thoroughly, the flasks and their contents were placed in an ultrasonic bath. After the polymer dissolved, ~10 g of 1,2,4-trimethylbenzene (TMB) was added to the mixtures as an expander initiator. The reaction solution was placed in a thermostatic water bath (approx. 35 °C) with a mechanical stirrer (rotation speed: 500 rpm) for 45 min. Subsequently, 35 g of TEOS was gradually added to the solution as a source of silica. The mixtures were left for 20 h under the given conditions. In the next step, the reaction mixtures were poured into the Teflon insert of the autoclave and heated to a temperature (80 °C or 100 °C) for one day. At this stage, the precipitates were aged in the initial solution. The precipitates obtained were filtered using a Büchner funnel, washed with distilled water (10 L), and dried at room temperature. Finally, the materials were calcined in a muffle furnace at 510 °C for 6 h.

Before synthesizing the hydrogels, the MCF-like silica materials were dried at 120 °C to remove physically bound water. Then, the chitosan biopolymer was dissolved in 2% acetic acid. The ratio of the components was fixed at 2 g of biopolymer per 100 mL of acetic acid solution. The resulting solution was stirred for 20 min, using a mechanical stirrer (500 rpm), in a water bath, at a temperature not exceeding 40 °C. After this time, 10 g of MCF silica was added, and the speed of the mixer was increased to 1000 rpm. The mixture was left under the given conditions for 24 h to obtain a homogeneous mixture. Finally, the flask with the contents was placed in a muffle furnace heated to 60 °C for two days to obtain the final material.

### 4.3. Methods and Calculations

#### 4.3.1. Measures Porosity and Pore Size Distributions

The textural properties of hydrogels were estimated from low-temperature nitrogen adsorption–desorption isotherms measured at 77 K over the range of relative pressures from 0 to 950 mmHg, using an analyzer (ASAP 2020, Micromeritics, Norcross, CA, USA). The specific surface area (S_BET_) was calculated from the experimental isotherms by using the standard BET method. The pore size distribution curves were determined from the adsorption and desorption branches of the isotherm, using the Barrett–Joyner–Halenda (BJH) model with cylindrical pores and the Faas correction. The total pore volume (V_t_) was determined from the amount of adsorbed nitrogen at p/p_0_ = 0.99. The pore volume of the micropores (V_mic_) was estimated using the t-plot method. Before the analysis, all samples were degassed at 60 °C and a pressure of 1 mmHg for 24 h in a degassing port of the analyzer. SAXS analysis was performed by X-ray diffraction (XRD) with an Empyrean diffractometer (PANalytical, Malvern, UK) with Cu anode X-ray tube radiation, using the SAXS/WAXS sample stage with capillary mode. The device was operated with a generator setting of 40 kV and 40 mA. The incident beam path consisted of W/Si, a stepped X-ray mirror with an elliptical shape. The SAXS measurements were performed at −0.1–4 degrees 2θ, with a step size of 0.005. The primary beam was measured with a Cu 0.2 mm beam attenuator and a PIXcel3D detector. The length of the scattering vector (or scattering vector), q, is defined as q = (4πsinθ)/λ, where 2θ is the scattering angle, and λ is the X-ray wavelength (1.5418 Å). Background scattering was performed by an air-scattering measurement, using an empty sample holder with foil. The Dv(R) calculations were performed using the indirect Fourier-transform technique applied in the EasySAXS software (PANalytical, Malvern, UK, version 1.2). In this case, the algorithm used is based on the Tikhonov regularization method.

#### 4.3.2. Potentiometric Titration

The acid–base properties of the hydrogel surface were determined based on the potentiometric titration measurements. The acidified suspension of a given hydrogel (NaCl electrolyte as a diluent) placed in the thermostatic vessel (25 °C) was titrated with NaOH solution, using an automatic burette (Dosimat 765, Metrohm, Herisau, Switzerland) connected with a pH-meter (PHM240, Radiometer, Copenhagen, Denmark). Based on the pH changes of a suspension in a titrant volume function, the surface charge densities and values of the point of zero charge for hydrogels were determined.

#### 4.3.3. Imaging the Microstructure and Morphology of the Materials by Electron Microscopy

The scanning electron microscopic (SEM) analysis was performed with the QuantaTM 3DFEG (FEI Company, Hillsboro, OR, USA) device at 5 kV. A high vacuum (4 × 10^−4^ Pa) was used to image the analyzed hydrogel samples. Before the SEM measurements, the samples were coated with gold to improve their electrical conductivity and their ability to reflect electrons and thus provide clear images. Atomic force microscopy (AFM) analysis was performed for the illustration 3D morphologies of one selected sample on a Bruker-Veeco-Digital Instruments Multi-Mode Atomic Force Microscope (Bruker, Bremen, Germany). The dynamic mode (tapping) was applied during AFM imaging. The NanoScope Analysis software, version 1.40 delivered from Bruker (Bruker, Bremen, Germany) was applied for the data treatment. Transmission electron microscope images (TEM) were collected on the Tecnai G2 T20 X-TWIN transmission microscopy.

#### 4.3.4. Analysis of the Elemental Composition

The carbon, hydrogen, and nitrogen analysis of the chitosan–silica hydrogels was carried out with the Series II CHNS/O analyzer 2400 (Perkin Elmer, Waltham, MA, USA). The temperature of the reduction and combustion processes was 950 °C. A total of 500 mg of each sample was used for the analysis.

#### 4.3.5. FTIR Mapping Tests

FTIR mapping analyses were performed with the FTIR iN10 MX microscope (Thermo Scientific, Waltham, MA, USA), using the specular reflectance method at room temperature, using the liquid nitrogen-cooled MCT-A detector.

#### 4.3.6. Adsorption at Equilibrium State

The adsorption of Acid Red 88 on chitosan–silica hydrogels was carried out using the static method with UV–Vis spectrophotometric measurements (Cary 4000, Varian Inc., Melbourne, Australia). In detail, 0.05 g of adsorbent was added to Erlenmeyer flasks and poured with dye solutions in a wide concentration range (0.04–1.96 mmol/L). The suspension solutions were placed in a thermostatic shaker (25 °C; 120 rpm) (Innova 40R model, New Brunswick, NJ, USA) for 48 h and then measured spectrophotometrically (after centrifugation). The equilibrium concentrations of the dye solutions were determined from absorption spectra at a wavelength of 503 nm. The amounts of dye adsorbed per 1 g of chitosan–silica hydrogel were calculated using the mass balance equation. The experimental adsorption isotherms were optimized using the Generalized Langmuir isotherm equation (GL) (Table 7).

#### 4.3.7. Adsorption Kinetics

The kinetics of the adsorption of Acid Red 88 on chitosan–silica hydrogels was investigated by spectrophotometric measurements (UV-Vis spectrophotometer Cary 100, Varian, Melbourne, Australia). The measurements were performed using the technique of continuous recording of absorption spectra in the wavelength range of 200–800 nm. In detail, 100 mL of dye solution with a concentration of 0.076 mmol/L was contacted with 0.05 g of a solid in a thermostatic glass vessel (Thermostat Ecoline RE 207, Lauda, Germany). During the experiment, the suspension solution was mechanically stirred (110 rpm), and, at a given time, a sample of the dye solution (after filtration) was directed to the measuring cell, measured, and returned to the reaction vessel. These activities were carried out automatically, using a flow cell and the special Cary instrument software, version 2 (Varian, Melbourne, Australia). The recorded absorption spectra were used to determine the concentration–time profiles, which were then optimized using the multi-exponential equation (m-exp) and the fractal-like MOE equation (f-MOE) (Table 8).

## Figures and Tables

**Table 1:** The values of the texture parameters that describe the porous structure of chitosan–silica hydrogels.

Hydrogel	S_BET_^a^ (m^2^/g)	S_ext_^b^ (m^2^/g)	V_t_^c^ (cm^3^/g)	V_mic_^d^ (t-plot) (cm^3^/g)	D_h_^e^ (nm)	D_BJH ads_^f^ (nm)	D_BJH des_^g^ (nm)
Ch_MCF1	351	318	0.65	0.01	7.4	7.9	4.2
Ch_MCF2	305	279	0.75	0.01	10.0	10.3	4.6
Ch_MCF3	181	173	0.31	0.00	7.0	7.2	5.7
Ch_MCF4	206	192	0.36	0.01	7.1	7.4	4.3

^a^ BET surface area calculated using experimental points at a relative pressure of (p/p_0_) 0.035–0.31, where p and p_0_ are denoted as the equilibrium and saturation pressure of nitrogen. ^b^ External surface area. ^c^ Total pore volume calculated by 0.0015468 amount of nitrogen adsorbed at p/p_0_ = 0.99. ^d^ Pore volume of micropores calculated by t-plot method with fitted statistical thickness in the range of 3.56 to 4.86 Å. ^e^ Hydraulic pore diameters calculated from the BET surface areas and pore volumes according to the equation: D_h_ = 4 V/S. ^f^ The pore diameter estimated from PSD maximum of BJH theory from adsorption data. ^g^ The pore diameter by BJH theory from desorption data.

**Table 2:** Physicochemical characteristics of the adsorbate.

Adsorbate	Formula	M (g/mol)	pK_a_	S (g/L)	D_max_ (nm)
AR88	C_20_H_13_N_2_NaO_4_S	400.4	11 [45]	1.5 [46]	1.40 [47]

M—molecular weight; S—solubility; D_max_—the distance between the most remote atoms in a dye molecule (Marvin program 14.8.25.0)

**Table 3:** The parameters of the Generalized Langmuir equation for the AR88/hydrogel systems.

Hydrogel	a_m_ (mmol/g)	m = n	log K	R^2^	SD (a)
Ch_MCF1	0.56	1	2.12	0.97	0.038
Ch_MCF2	0.59	1	2.18	0.92	0.062
Ch_MCF3	0.55	1	1.68	0.84	0.081
Ch_MCF4	0.63	1	2.04	0.94	0.061

**Table 4:** Comparison of the maximum adsorption capacity of various adsorbents towards AR88.

Adsorbent (pH)	Maximum Adsorption Capacity, a_m_ (mmol/g)	Ref.	Integral Cost–Benefit
Surfactant modified bentonite (neutral)	0.23	[31]	High
Chitosan–silica gel composite (neutral)	0.48	[32]	Low
Chitosan–nanosilica composite (pH 2)	0.75	[33]	Moderate
Chitosan–nanosilica composite (neutral)	0.51	[33]	Moderate
Magnetic ZnFe_2_O_4_ spinel ferrite nanoparticles (pH 3.2)	0.33	[34]	High
ZnO/ZnMn_2_O_4_ nanocomposite (neutral)	0.19	[35]	High
Magnetic MWCN-Fe_3_C composite (neutral)	0.14	[36]	High
Zeolite–chitosan hydrogel (neutral)	1.02	[37]	Low
Chitosan–silica hydrogel (neutral)	0.63	This paper	Low

**Table 5:** Comparison of the selected parameters determined from the multi-exponential equation and the fractal-like MOE equation.

Hydrogel	fit	f_2_/p	log k*	t_0.5_ (min)	t_75%_/t_90%_ (min)	u_eq_	SD(c/c_0_) [1]	1-R^2^
Ch_MCF1	m-exp	-	−0.78	4.14	24.5/78	0.98	0.85	2.35·10^−3.^
	f-MOE	−0.16/0.4	−0.89	4.03	23/90	0.98	1.57	4.83·10^−3^
Ch_MCF2	m-exp	-	−0.17	1.03	2.6/10.5	0.99	0.96	1.67·10^−2^
	f-FOE	0/0.47	−0.16	0.66	3.1/10	0.98	0.61	1.03·10^−3^
Ch_MCF3	m-exp	-	−0.84	4.85	70/440	0.94	1.08	4.35·10^−3^
	f-SOE	1/0.46	−0.78	6.05	62/690	1	1.55	5.89·10^−3^
Ch_MCF4	m-exp	-	−1.21	11.23	52/144	0.98	1.18	3.29·10^−3^
	f-MOE	−0.46/0.4	−1.18	11.59	50/158	1	1.86	6.25·10^−3^

**Table 6:** Details and synthesis conditions of MCF-type silica and chitosan–silica hydrogels and summary of elemental analysis results for chitosan–silica hydrogels.

Hydrogel	Reagents and Conditions for Silica Phase			Chitosan (g)	Composition		
	Polymer Type	TMB (mL)	Ageing Temperature (°C)		%C	%H	%N
Ch_MCF1	PE10500	10.7	80	2	7.1	1.9	0.8
Ch_MCF2	PE9400	10.8	80	2	7.9	2.4	1.1
Ch_MCF3	PE10500	10.2	100	2	9.1	2.2	1.1
Ch_MCF4	PE9400	10.6	100	2	7.9	2.1	1.2

**Table 7:** Theoretical isotherm equation used to optimize the adsorption data.

Isotherm	Equation	Isotherm	Equation	m	n
Generalized Langmuir (GL)	$\theta ={\left[\frac{{\left(Kc_{eq}\right)}^{n}}{1+{\left(Kc_{eq}\right)}^{n}}\right]}^{m/n}$	Langmuir–Freundlich (LF)	$\theta =\frac{{\left(Kc_{eq}\right)}^{m}}{1+{\left(Kc_{eq}\right)}^{m}}$	$\in \left(0,\left.1\right)\right.,$ m = n	$\in \left(0,\left.1\right),\right.$ m = n
		Generalized Freundlich (GF)	$\theta ={\left[\frac{Kc_{eq}}{1+Kc_{eq}}\right]}^{m}$	$\in \left(0,\left.1\right)\right.$	1
		Tóth (T)	$\theta =\frac{Kc_{eq}}{{\left[1+{\left(Kc_{eq}\right)}^{n}\right]}^{1/n}}$	1	$\in \left(0,\left.1\right)\right.$
		Langmuir (L)	$\theta =\frac{Kc_{eq}}{1+Kc_{eq}}$	1	1

where *θ* is the global adsorption isotherm, *m* and *n* are the heterogeneity parameters, and *K* is the adsorption equilibrium constant.

**Table 8:** Theoretical equations used to optimize the kinetic data.

Kinetic Model	General Equation	Half-Time Expression
Multi-exponential equation (m-exp)	$c=\left(c_{o}-c_{eq}\right)\sum_{i=1}^{n}f_{i}exp\left(-k_{i}t\right)+c_{eq}$	*t*_0.5_*~*(ln 2)/k_i_
Fractal-like MOE equation (f-MOE)	$F=\frac{{1-exp\left(-k_{1}t\right)}^{p}}{{1-f_{2}exp\left(-k_{1}t\right)}^{p}}$	*t*_0.5_~[ln(2-f_2_)]^1/p^/k_1_
Fractal-like FOE equation (f-FOE)	$F={1-exp\left(-k_{1}t\right)}^{p}$	*t*_0.5_~[(ln2)]^1/p^/k_1_
Fractal-like SOE equation (f-SOE)	$F=\frac{{\left(k_{2}t\right)}^{p}}{{1+\left(k_{2}t\right)}^{p}}$	*t*_0.5_~1/k_2_

where *c_eq_* is the equilibrium concentration, *c_o_* is the initial concentration, *c* is the temporary concentration, *k* is the kinetic rate coefficient, *t* is time, *F* is the adsorption progress, and *p* is the fractal parameter.

## Data Availability

The original contributions presented in the study are included in the article, further inquiries can be directed to the corresponding authors.
